# Long-term adjuvant administration of temozolomide impacts serum ions concentration in high-grade glioma

**DOI:** 10.1186/s41016-022-00271-7

**Published:** 2022-02-25

**Authors:** Liyun Zhong, Pei Yang, Chuanbao Zhang, Zheng Wang, Tao Jiang, Baoshi Chen, Xia Shan, Xiaoguang Qiu

**Affiliations:** 1 Department of Neurosurgery, Beijing Tiantan Hospital, Capital Medical University, Beijing, China; 2 Department of Molecular Neuropathology, Beijing Neurosurgical Institute, Capital Medical University, Beijing, China; 3 Chinese Glioma Genome Atlas Network (CGGA) and Asian Glioma Genome Atlas Network (AGGA), Beijing, China; 4 Center of Brain Tumor, Beijing Institute for Brain Disorders, Beijing, China; 5 China National Clinical Research Center for Neurological Diseases, 119 South 4th Ring Road West, Beijing, 100070 China; 6 Department of Radiotherapy, Beijing Tiantan Hospital, Capital Medical University, 119 South 4th Ring Road West, Beijing, 100070 China

**Keywords:** High-grade glioma (HGG), Temozolomide (TMZ), Chemotherapy, Toxicity

## Abstract

**Background:**

Adjuvant temozolomide (TMZ) chemotherapy with standard regimen remarkably improves survival in patients with high-grade glioma (HGG). However, the influence of long-term TMZ chemotherapy on serum ions concentration is unclear.

**Methods:**

One hundred and thirty-eight patients with HGG were included. Their blood samples were collected for blood biochemistry and routine test. The alteration in serum ions concentration, total protein, albumin, globin, and blood cells counts were used to identify the impact of long-term TMZ chemotherapy.

**Results:**

Through the comparation of quantitative value of diverse parameters among different chemotherapy cycles, we identified that serum potassium concentration had a downward trend after TMZ administration (1st vs. 6th, *p* < 0.001; 1st vs. 12th, *p* < 0.001). Additionally, the correlation analysis showed that platelets was negatively correlated with chemotherapy cycles (*r* = − 0.649, *p* = 0.023). The hematological adverse events mainly centered on grade 1 to 2.

**Conclusion:**

Long-term administration of TMZ may lead to serum ions disturbance. Besides the myelosuppression, we should pay attention to the alteration in serum ions concentration, and give patients proper symptomatic treatment when necessary.

**Supplementary Information:**

The online version contains supplementary material available at 10.1186/s41016-022-00271-7.

## Background

As the most common intracranial primary malignant tumor in adult, glioma is characterized by high invasiveness and recurrence rate, especially for high-grade glioma (HGG, grade III and IV) [1, 2]. Median survival of HGG patients remain merely 12–17 months despite of the standard treatment strategy—maximal safe resection with postoperative radiochemotherapy [3, 4]. Temozolomide (TMZ), as an oral imidazotetrazine family alkylator, can methylate DNA, which most often occurs at the N-7 or O-6 position of guanine residues. This methylation damages the DNA and triggers the death of tumor cells. Approved by Food Drug Administration in 1999, TMZ was used as a second-generation alkylating agent in treating gliomas [5]. Because of the efficacy and tolerance, TMZ became a first-line treatment for glioblastoma (GBM) patients [6]. In the profound EORTC-NCIC study, the treatment strategy of postoperative radiotherapy with concurrent and adjuvant TMZ was well established. Even for elderly patients receiving adjuvant hypofractionated radiotherapy, the adjuvant TMZ also showed efficacy on survival benefit [7, 8]. For patients with O6-methylguanine DNA methyltransferase (MGMT) promoter methylation, the sensitivity of TMZ was enhanced, and survival increased strikingly. In other studies, MGMT promoter methylation was verified to be an independent prognostic factor of glioma patients. Conversely, high expression level of MGMT was related to the resistance of TMZ and shorter overall survival [9]. Considering the toxicity of TMZ, previous studies had reported that fatigue was a common side effect in the majority (33–76%), and other side effects including digestive tract side effects, elevated liver enzymes, lymphocytosis and myelosuppression, etc. [6, 10]. However, most of the adverse events could be alleviated by systematic treatment and very little deadly adverse events [11]. Presently, some studies reported that LGG could relapse to become HGG and the hypermutation was observed in tumor after receiving TMZ chemotherapy, which promoted the hypothesis of TMZ-induced hypermutation [12, 13].

Postoperative radiotherapy with concurrent and adjuvant TMZ chemotherapy is universally recognized in clinical practice [7]. In recent years, a great number of researches have emerged around TMZ chemotherapy. Not only focusing on the efficacy of diverse TMZ regimens, such as dose-dense regimen and continuous low-dose regimen, studies also discussed the appropriate choice of chemotherapy cycles [14, 15]. The selection of TMZ chemotherapy cycle remains controversial. Some retrospective studies suggested that prolonging the chemotherapy cycle (> 6 cycles) could improve patients’ progression-free survival and overall survival [16–18]. While others observed no significant benefit from long-term TMZ chemotherapy [19, 20].

Besides the effect on survival, there were studies assessing the influence of cisplatin on serum ion concentration and trace elements [21, 22]. However, rare studies focus on the effect of TMZ chemotherapy on serum ion concentration in patients with glioma. Serum ions such as potassium, calcium and sodium, etc., play important roles in maintaining human electrolyte balance, acid and alkali balance and normal cell function. TMZ, as a small-molecule drug, can be absorbed by digestive tract and enter blood circulation to take effect in the lesion. Meanwhile, TMZ may affect the serum ion concentration and further minimize the tolerance. Therefore, in this study, we aim to explore the side-effect of TMZ on serum ion concentration. Assessing the changes in blood biochemistry and blood routine test of HGG patients during TMZ chemotherapy will provide us a comprehensive understanding of TMZ and a better idea of balancing the efficacy and toxicity of TMZ.

## Methods

### Patients and data collection

There were two cohorts in this study. The first cohort included 73 recurrent HGG samples with postoperative chemotherapy information and RNA sequencing data available (48 with postoperative chemotherapy and 25 without) (Table 1). The data were downloaded from China Glioma Genome Atlas (CGGA) (http://www.cgga.org.cn).

**Table 1:** The clinical characteristics of patients in CGGA dataset

Factors		Number of patients
**Cohort 1**		**73**
Age	≤ 50	60
	> 50	13
Gender	Male	49
	Female	24
Grade	III	23
	IV	50
Chemotherapy	Yes	48
	No	25
Radiotherapy	Yes	31
	No	39
	Unknown	3
**Cohort 2**		**138**
Age	≤ 50	72
	> 50	66
Gender	Male	88
	Female	50
Grade	III	54
	IV	84
KPS (before TMZ)	≤ 70	18
	> 70	77
	Unknown	43

*KPS* Karnofsky performance score

In the second cohort, two hundred and eighteen HGG patients diagnosed with the World Health Organization (WHO) grade III and IV primary gliomas from Beijing Tiantan Hospital during May 2011 to March 2018 were enrolled in this study. Considering the large variation of time interval between two chemotherapy cycles in patients, patients with interval time more than 56 days were excluded. One hundred and thirty-eight postoperative patients were included finally. All patients received adjuvant TMZ (150–200 mg/m^2^/day for days 1–5 every 28 days) in our hospital. Seventy-three patients received at least 12 cycles of TMZ chemotherapy while 28 patients received at least 18 cycles. The range of chemotherapy cycles was from two to thirty-seven. After 6-cycle standard adjuvant chemotherapy, extended chemotherapy was performed according to the tumor residual and patients’ tolerance. The blood samples were collected before the next cycle of chemotherapy. Testing items included blood biochemistry (calcium, Ca; magnesium, Mg; iron, Fe; potassium, K; sodium, Na; chloride, Cl; total protein, TP; albumin, Ab; globulin, Gb) and blood routine (white cell count, WBC; red cell count, RBC; hemoglobin, HGB; platelet count, PLT) (Table S1). The characteristics of patients were showed in Table 1. This study was approved by the Ethics Committee of Capital Medical University, Beijing, China.

### Toxicity assessment

The Common Terminology Criteria for Adverse Events (CTCAE) version 5.0 established by the National Cancer Institute was applied to assess the severity of the hematologic toxicity for patients in the second cohort. In CTCAE system, grade 1 to 5 indicates mild adverse effects, moderate adverse effects, severe or medically significant adverse effects, life-threatening adverse effects and death related to adverse effects, respectively. Here, the degree of thrombocytopenia, leukopenia, anemia, and erythropenia were evaluated by using CTCAE criteria (Table S2).

### Statistical analysis

R language was used for statistical analysis and generating figures (https://www.r-project.org/). Public packages “ggplot2,” “fgsea,” “qusage,” and “pheatmap” were used to perform statistical computation. Gene set enrichment analysis (GSEA) was applied to annotate the function of genes that were highly expressed in the chemotherapy group in the first cohort. In the second cohort, Spearman rank correlation analysis was applied to detect the correlation between chemotherapy cycles and the change of parameters. In patients with the same chemotherapeutic cycles, analysis of paired t test or paired Wilcoxon symbolic rank test was used to compare the change of ions concentration between different periods of chemotherapy. Chi-square test was used to compare the difference between hematological adverse events and chemotherapy cycles. In all statistical analysis, a *p* < 0.05 was considered significant.

## Results

### TMZ chemotherapy could affect the ion channel associated expression profiles

**Fig. 1:**
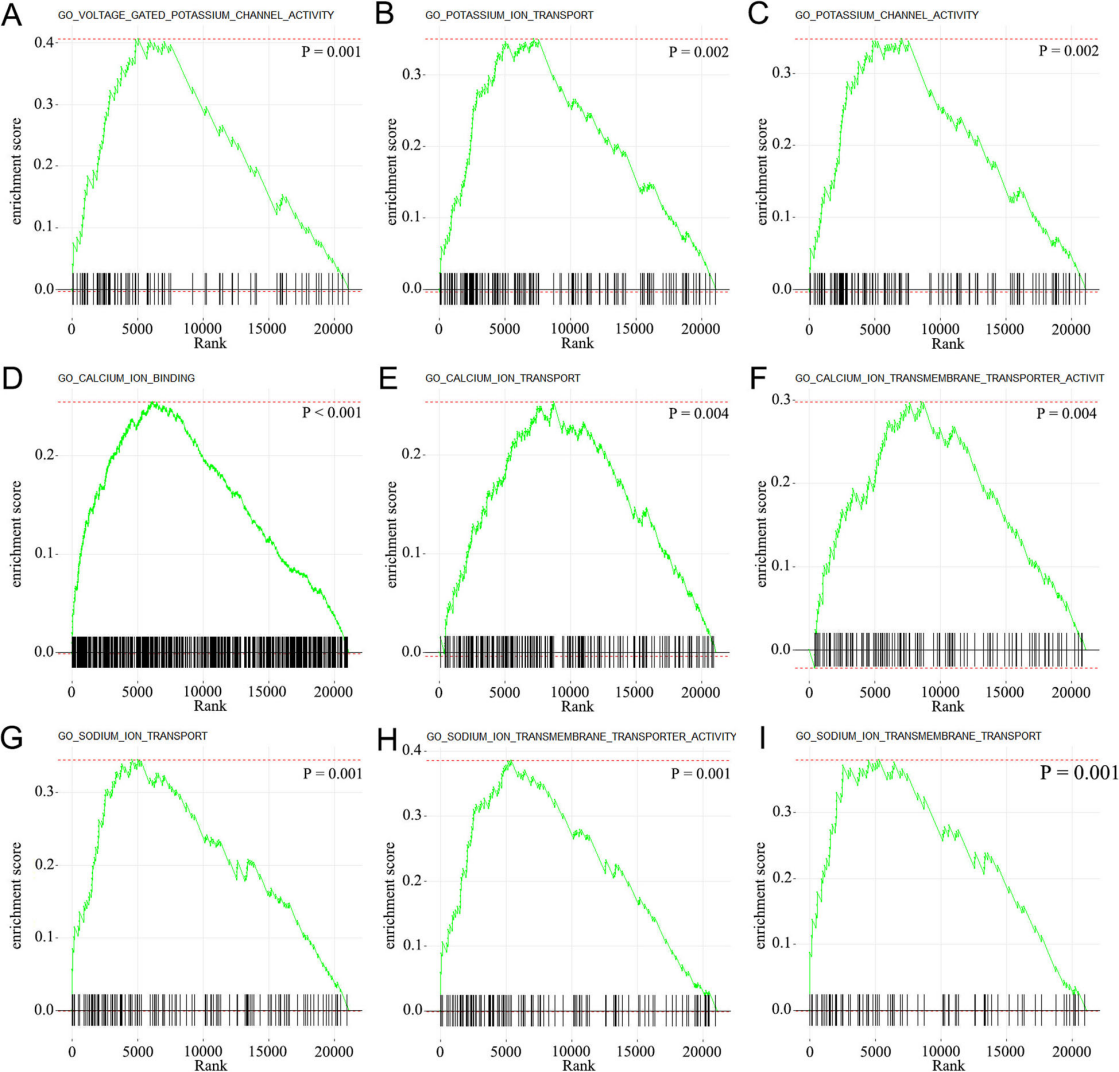
The result of GSEA analysis showed that the function of differentially expressed genes between chemotherapy and non-chemotherapy enriched in ion channels. **A**–**C** Potassium ion channel related signaling pathways. **D**–**F** Calcium ion channel-related signaling pathways. **G**–**I** Sodium ion channel related signaling pathways

To investigate the effect of TMZ chemotherapy on the expression profiles of HGG samples, we downloaded the RNA sequencing data of 73 recurrent HGGs with postoperative chemotherapy information available from CGGA database (Table 1). Patients were divided into two groups according to the chemotherapy status after the first surgery (48 with postoperative chemotherapy and 25 without). GSEA analysis was applied to characterize the differentially expressed genes between the two groups. The results showed that the most differently expressed genes (777 genes, *p* < 0.05, log2(fold change) > 1) were enriched in ion channels and ion transmembrane transport, including calcium, potassium, sodium, and chloride channels (Fig. 1, all *p* < 0.01).

### Prolonged TMZ chemotherapy could affect the serum ion concentration

**Fig. 2:**
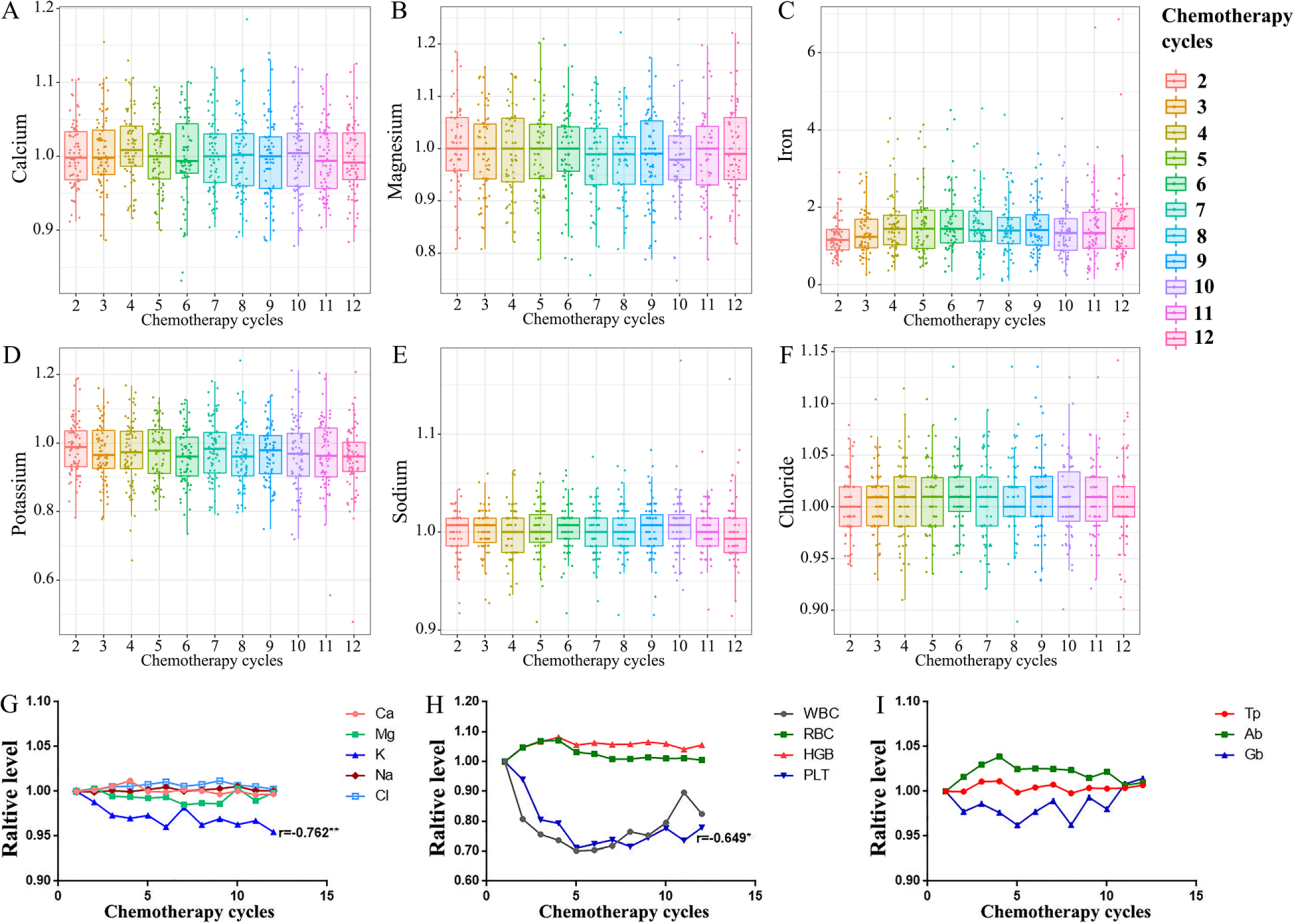
**A**–**F** The variation trend of serum ions concentration with the increase of chemotherapy cycles. **G**–**I** The correlation analysis between serum parameters and chemotherapy cycles

To validate the effect of TMZ chemotherapy on the concentration of serum ions, we collected the blood test results (blood biochemistry and blood routine test) from 138 patients in the second cohort before every cycle of TMZ chemotherapy. To reduce the variation of the baseline levels in each individual, we used their first blood test value as a baseline, and focused on the relative level (ratio of the following values compared with the initial value) of the ions. With the increase of TMZ chemotherapy cycles, serum ions concentration showed slight fluctuations (Fig. 2A–F). The correlation analysis showed that serum potassium concentration was negatively correlated with the cycle of chemotherapy (*r* = − 0.762, *p* = 0.004; Fig. 2G), while serum iron concentration showed positive correlation (*r* = 0.747, *p* = 0.005; Fig. S1). There was no significant correlation between the chemotherapy cycles and the change of the relative levels of calcium, magnesium, sodium and chlorine (all *p* > 0.05). To detect the difference more directly, we compared the quantitative value of the ions in different chemotherapy cycles (Table 2). The results showed that serum potassium concentration had a downward trend after TMZ administration (1st vs. 6th, *p* < 0.001; 1st vs. 12th, *p* < 0.001). The serum sodium concentration diminished slightly between the sixth cycles and the twelfth cycle (*p* = 0.029). While, an elevated serum concentration was observed in certain ions, such as iron (1st vs. 6th, *p* < 0.001; 1st vs. 12th, *p* < 0.001) and chlorine (1st vs. 6th, *p* = 0.009). Therefore, we observed definite correlations between TMZ chemotherapy and ion concentrations in HGG patients.

**Table 2:** The comparison of ion concentrations in different chemotherapy cycles

Parameters	Mean ± SD	Comparison of different cycles	***P*** value	Mean ± SD	***P*** value
**Calcium (mmol/L)**	1st: 2.35 ± 0.11	1st vs 6th	0.964	1st: 2.35 ± 0.10	**0.043**
	6th: 2.34 ± 0.12	1st vs 12th	0.507	18th: 2.290.10	
	12th: 2.34 ± 0.10	6th vs 12th	0.354		
**Magnesium (mmol/L)**	1st: 0.91 ± 0.07	1st vs 6th	0.769	1st: 0.92 ± 0.08	0.394
	6th: 0.90 ± 0.07	1st vs 12th	0.846	18th: 0.91 ± 0.75	
	12th: 0.91 ± 0.07	6th vs 12th	0.871		
**Iron (μmol/L)**	1st: 14.89 ± 8.72	1st vs 6th	**< 0.001**	1st: 17.10 ± 12.10	0.280
	6th: 20.08 ± 6.12	1st vs 12th	**< 0.001**	18th: 17.71 ± 5.54	
	12th: 19.42 ± 7.00	6th vs 12th	0.307		
**Potassium (mmol/L)**	1st: 4.38 ± 0.35	1st vs 6th	**< 0.001**	1st: 4.30 ± 0.36	**0.049**
	6th: 4.19 ± 0.31	1st vs 12th	**< 0.001**	18th: 4.11 ± 0.32	
	12th: 4.16 ± 0.41	6th vs 12th	0.479		
**Sodium (mmol/L)**	1st: 141.99 ± 2.71	1st vs 6th	0.056	1st: 141.40 ± 2.60	0.563
	6th: 142.58 ± 2.59	1st vs 12th	0.487	18th: 141.79 ± 2.97	
	12th: 142.03 ± 4.17	6th vs 12th	**0.029**		
**Chloride (mmol/L)**	1st: 104.92 ± 3.15	1st vs 6th	**0.009**	1st: 104.46 ± 3.23	0.253
	6th: 106.05 ± 3.10	1st vs 12th	0.601	18th: 105.39 ± 2.79	
	12th: 105.27 ± 4.52	6th vs 12th	0.085		

Bold numbers represent statistical significance

**Fig. 3:**
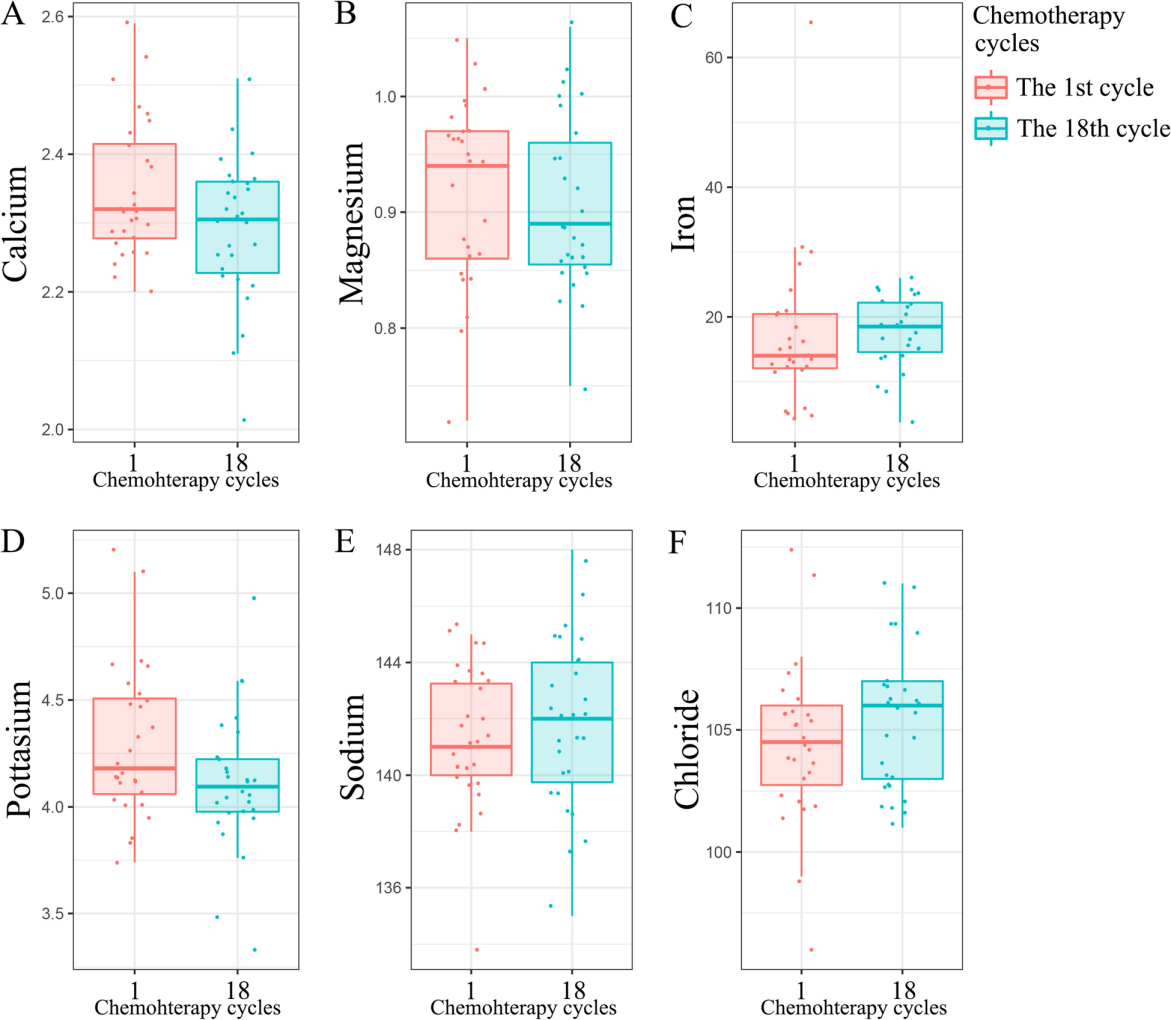
**A**–**F** The comparison of serum ions concentration changes between the first cycle of temozolomide chemotherapy and the eighteenth cycle

To further assess the accumulative effect of long-term TMZ treatment, we identified 28 patients who received at least 18 cycles of chemotherapy from the second cohort. The comparation between the first cycle and the eighteenth cycle showed the changing trend of serum ions concentration with prolonged chemotherapy cycles (Table 2). The serum concentration of calcium (*p* = 0.043) and potassium (*p* = 0.049) significantly declined after 18 cycles chemotherapy (Fig. 3A, D), while other parameters (magnesium, iron, sodium and chlorine) were not (Fig. 3B, C, E and F). Thus, we inferred that prolonged chemotherapy cycles might impact the serum ions homeostasis.

### The impact of prolonged TMZ chemotherapy on hematological toxicity and nutritional condition

According to the published adverse events of TMZ, myelosuppression is a common side effect, especially leukopenia and thrombocytopenia. In addition, gastrointestinal dysfunction, such as nausea and vomiting, is also common, which may lead to the decreased appetite and even bad nutritional status. In the present study, we focused on the alteration of such parameters after long-term administration of TMZ. Seventy-three patients received at least 12 cycles of TMZ chemotherapy of cohort two was applied for analysis. The relative level of PLT showed negative correlation with TMZ chemotherapy (*r* = − 0.649, *p* = 0.023) (Fig. 2H) while other parameters (WBC, RBC, Tp, Ab, and Gb) not (Fig. 2H, I). According to the CTCAE criteria, the hematological adverse events mainly centered on grade 1 to 2, and none of patients suffered from grade 3–5 side effects (Table S3). The number of patients with higher grade of side effects was elevated with increased chemotherapy cycles (leukopenia, *p* = 0.006; thrombocytopenia, *p* = 0.047) (Fig. S2). With increased chemotherapy cycles, higher-grade leukopenia and thrombocytopenia were increased, which indicated the elevated risk of myelosuppression after prolonging TMZ chemotherapy cycles.

## Discussion

The toxicity of TMZ has been widely investigated. Fatigue, gastrointestinal side effects, leukopenia and thrombocytopenia were recognized as common adverse events [11, 23]. To our knowledge, rare studies focus on the change of serum ions concentration after TMZ chemotherapy in HGG patients. The alteration of serum ions concentration may impact the pharmacological mechanism of the drug, and may be related to the occurrence of adverse events [21, 24, 25]. In the present study, we applied RNA sequencing data and blood samples to analyze the influence of long-term TMZ chemotherpay on serum ions concentration.

According to the result of GSEA analysis, the function of differentially expressed genes was mainly involved in ion channels, such as potassium ion, calcium ion and sodium ion, etc. Compared with those without chemotherapy, patients received TMZ chemotherapy presented an up-regulation of genes associated with potassium, calcium and sodium ion transport, which reminded us that TMZ chemotherapy might change the serum ions concentration via regulating certain ion channels. To verify our findings, we included eligible patients with HGG and collected their blood samples to further analysis. For those patients who received at least 12 cycles chemotherapy, their serum ions concentration and the hematological parameters tended to fluctuate. The correlation analysis revealed that serum potassium concentration and PLT were decreased visibly with the increase of chemotherapy cycles. *T* test and Wilcoxon signed-rank test were applied for analyzing the differences among the first cycle, the sixth cycle and the eighteenth cycle chemotherapy. The results showed that the disorder of serum ions concentration was associated with long-term chemotherapy. However, except for some extremums, most indicators could maintain within normal range. Benefiting from the oral administration, patients did not need to stay in the hospital for a long time, which, on the other hand, affected the results to a certain extent. There were great differences in their dietary habit, digestive side effect as well as taking Chinese traditional medicine, which led to the inevitable biases. We attempted to enlarge the sample size to minimize such biases. Whereas, individual differences among patients could not be eliminated. Therefore, we performed data analysis to remind the clinicians paying attention to the risk of serum ions metabolism disorder after prolonged cycles of TMZ chemotherapy.

It has been reported that TMZ-induced cell death and apoptosis was mediated by O6-methylguanine (O6-meG) production and its reactive oxygen species production. TMZ-induced O6-meG production, involved in AMP-activated protein kinase (AMPK) activation, and contributed to cell apoptosis by promoting p53 activation and inhibiting mTORC1 signaling [26, 27]. Based on previous research, significant reduction in cell volume resulting from loss of potassium and chloride ion is a hallmark of cell apoptosis [28, 29]. Algharabli et al. investigated the mechanism of TMZ-mediated apoptosis in GBM cells. They identified that TMZ can trigger the loss of potassium and chloride ions through specific ion channel [30]. The activation of AMKP induced by TMZ may inhibit the intermediate-conductance calcium-activated potassium channel (KCa 3.1) [31]. What is more, using the patch-clamp technology, Yeh et al. detected the direct inhibitory effect of TMZ on KCa 3.1 [32]. Besides the calcium-activated potassium channel, the voltage-gated potassium channel, such as Kv10.1, was also verified to be correlated with poor prognosis in GBMs [33]. Thus, we inferred that TMZ might affect the serum ions concentration via regulating the expression of certain ion channels. The related experiments are need in the future to proof the presumption.

In addition, the interaction among ions metabolism should be concerned with. Some studies suggested that the maintenance of serum potassium concentration resulted in sustained improvement in calcium balance [34]. Approximately 50% of hypokalemia accompanied with hypomagnesemia. And hypomagnesemia may lead to severer and more refractory hypokalemia [35, 36]. Therefore, routine examination of blood biochemistry and blood routine are necessary for patients under TMZ chemotherapy. When the serum potassium reduced, supplying magnesium simultaneously may improve the effect of potassium supplement, and the serum concentration of calcium may also increase.

## Conclusions

Long-term TMZ chemotherapy may lead to the disturbance of ions concentration by influencing the specific ion channels. In addition to the focus on myelosuppression, we should pay attention to the alteration in serum ions concentration, and give patients proper symptomatic treatment when necessary. However, the dietary supplement, treatment before chemotherapy, anti-emetics and individual difference are unavoidable factors that may influence the results. Thus, well-designed large clinical trials and rigorous experiments are needed in the future.

## Supplementary Information

**Additional file 1: Figure S1.** The correlation analysis between serum parameters and chemotherapy cycles (with iron).

**Additional file 2: Figure S2.** Chi-square analysis between hematological adverse events and chemotherapy cycles. (A) Leukopenia; (B) Thrombocytopenia.

**Additional file 3: Table S1.** The normal ranges of blood routine and blood biochemistry test.

**Additional file 4: Table S2.** Hematological adverse event assessment in TMZ administration according to the CTCAE criteria.

**Additional file 5: Table S3.** The grade of adverse events according to CTCAE criteria.

## Data Availability

The datasets generated during and analyzed during the current study are available from the corresponding author on reasonable request.

## Abbreviations

TMZ: Temozolomide
HGG: High-grade glioma
GBM: Glioblastoma
MGMT: O6-methylguanine DNA methyltransferase
CGGA: China Glioma Genome Atlas WHO World Health Organization
CTCAE: The Common Terminology Criteria for Adverse Events
GSEA: Gene set enrichment analysis
AMPK: AMP-activated protein kinase
KCa 3.1: Intermediate-conductance calcium-activated potassium channel
