# Supplementary figures and images for: Long-term adjuvant administration of temozolomide impacts serum ions concentration in high-grade glioma
Source: Chin Neurosurg J. 2022 Feb 25;8:6. doi: 10.1186/s41016-022-00271-7 (PMC8876447; doi:10.1186/s41016-022-00271-7)

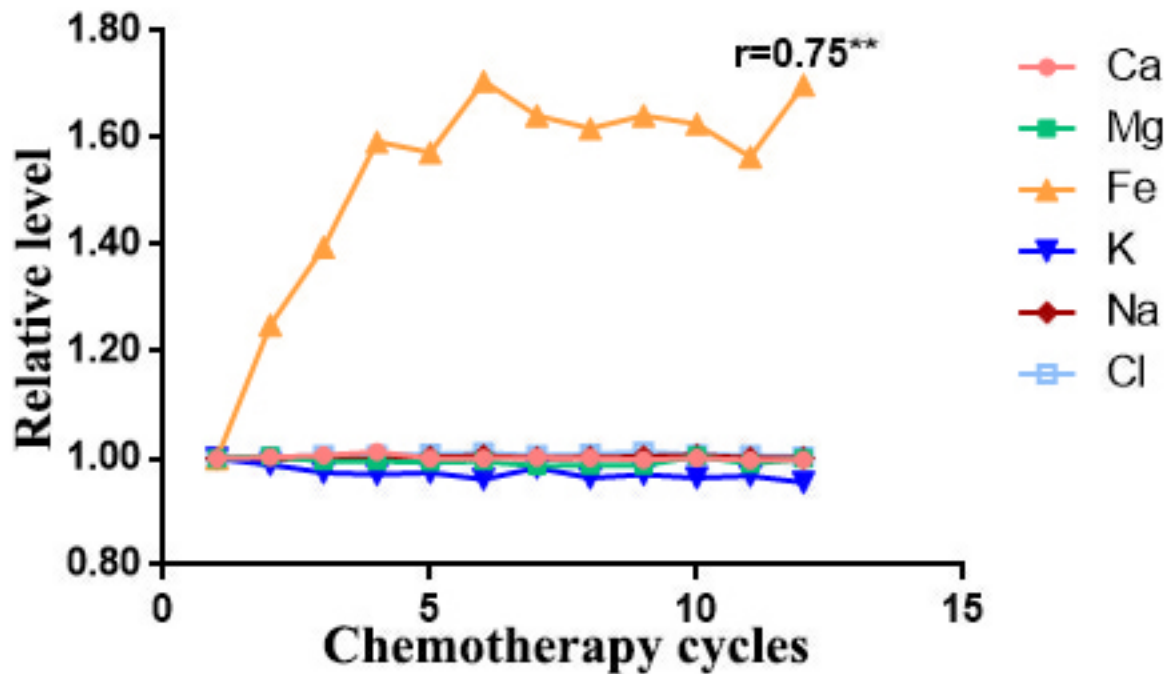

Supplement: Supplementary file 1 — Additional file 1: Figure S1. The correlation analysis between serum parameters and chemotherapy cycles (with iron). [file 41016_2022_271_MOESM1_ESM.pdf]

**A****Leukopenia****\*\***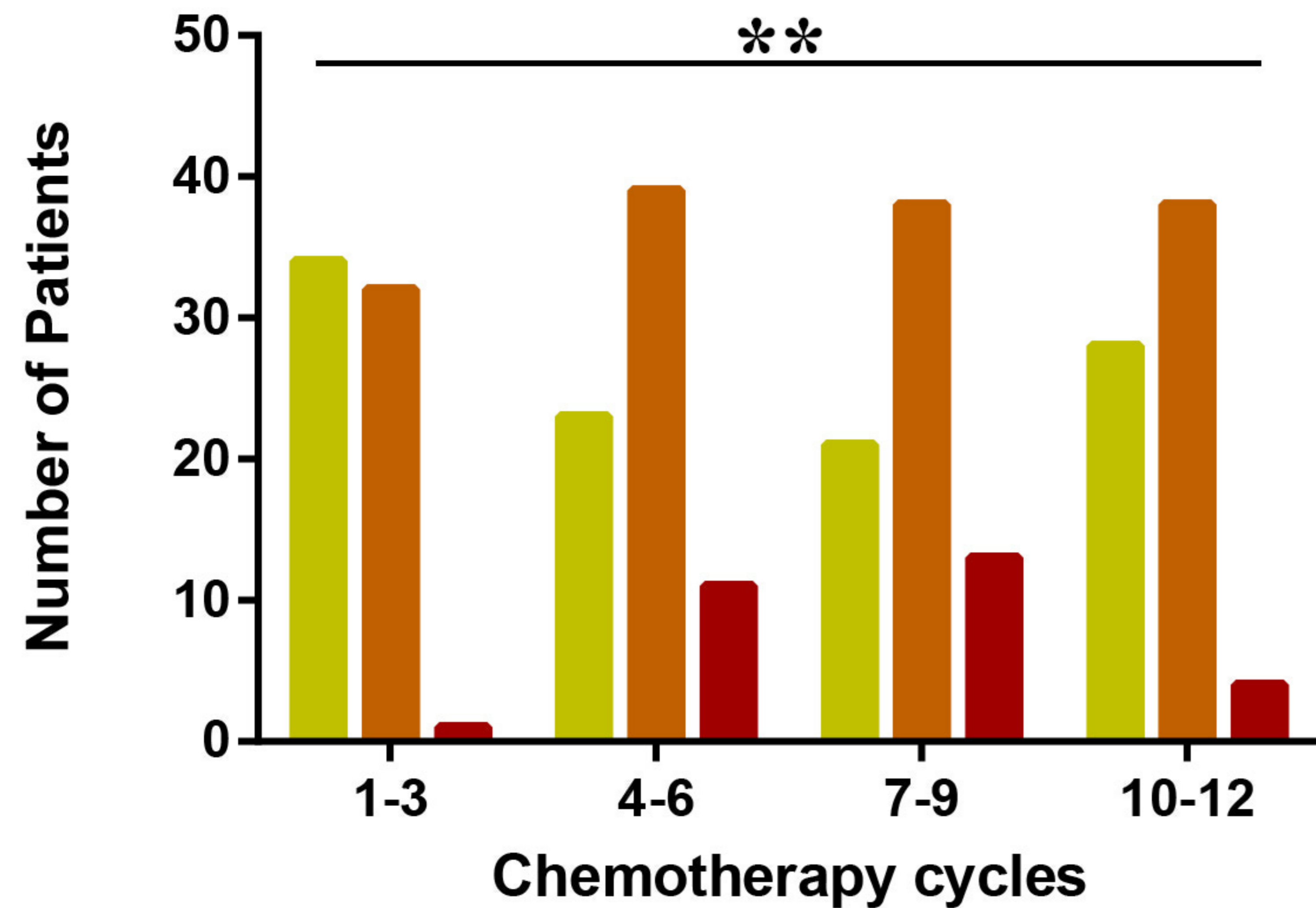**B****Thrombocytopenia****\***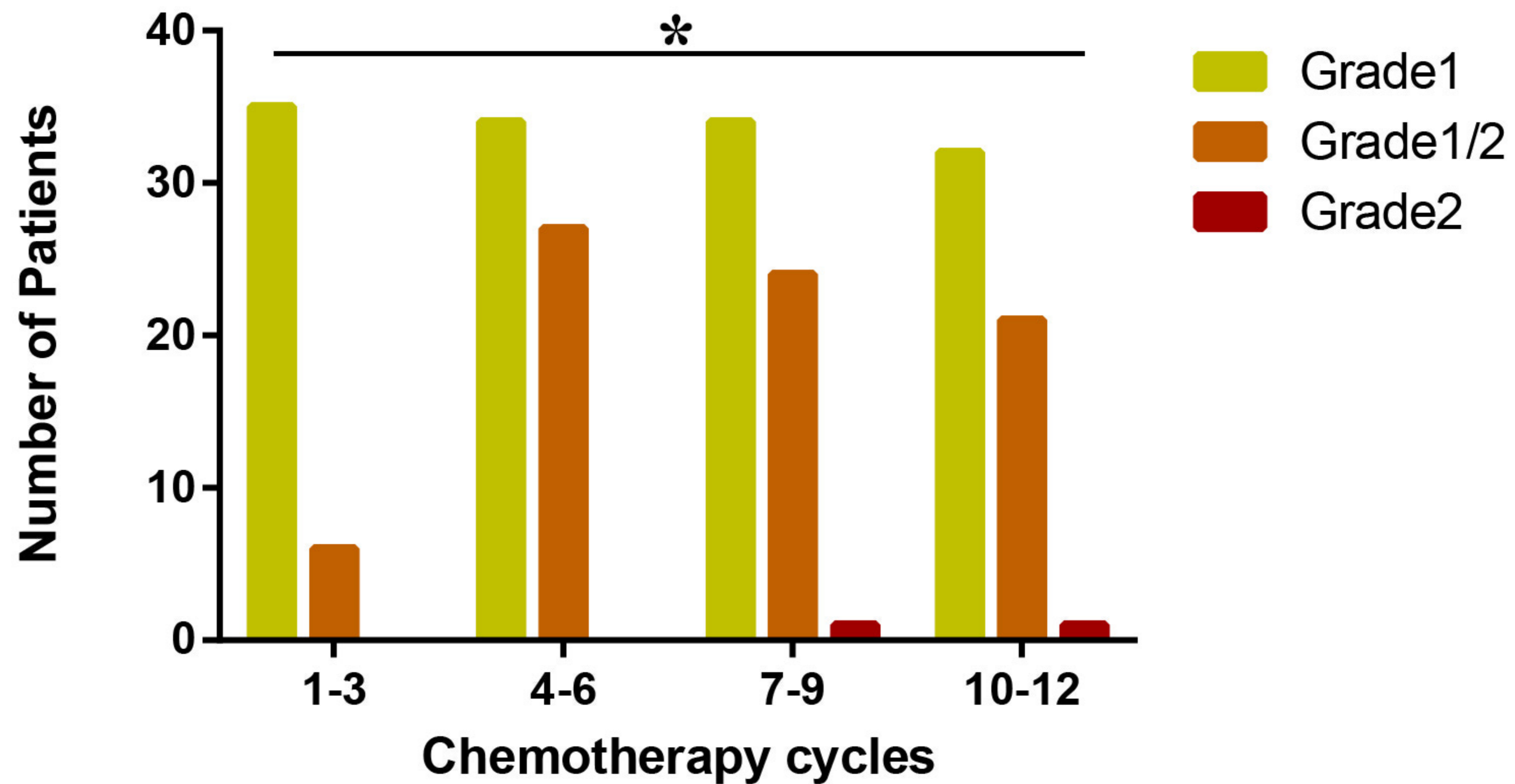

Supplement: Supplementary file 2 — Additional file 2: Figure S2. Chi-square analysis between hematological adverse events and chemotherapy cycles. (A) Leukopenia; (B) Thrombocytopenia. [file 41016_2022_271_MOESM2_ESM.pdf]
