# Supplementary material for: Long-term adjuvant administration of temozolomide impacts serum ions concentration in high-grade glioma
Source: Chin Neurosurg J. 2022 Feb 25;8:6. doi: 10.1186/s41016-022-00271-7 (PMC8876447; doi:10.1186/s41016-022-00271-7)
Supplement: Supplementary file 3 — Additional file 3: Table S1. The normal ranges of blood routine and blood biochemistry test. [file 41016_2022_271_MOESM3_ESM.docx]

**Table S1** The normal ranges of blood routine and blood biochemistry test.

| **Test index** | **Normal range** |
| --- | --- |
| White cell count | 3.5-9.5 (*10^9/L) |
| Red cell count | 4.3-5.8 (*10^12/L) |
| Hemoglobin | 130-175 (g) |
| Platelet count | 125-350 (*10^9/L) |
| Sodium | 137-147 (mmol/L) |
| Chloride | 99-110 (mmol/L) |
| Potassium | 3.5-5.3 (mmol/L) |
| Calcium | 2.2-2.7 (mmol/L) |
| Magnesium | 0.75-1.02 (mmol/L) |
| Iron | 10.6-36.7 (μmol/L) |
| Total protein | 65-85 (g/L) |
| Albumin | 40-55 (g/L) |
| Globulin | 20-40 (g/L) |
