# Supplementary material for: Long-term adjuvant administration of temozolomide impacts serum ions concentration in high-grade glioma
Source: Chin Neurosurg J. 2022 Feb 25;8:6. doi: 10.1186/s41016-022-00271-7 (PMC8876447; doi:10.1186/s41016-022-00271-7)
Supplement: Supplementary file 4 — Additional file 4: Table S2. Hematological adverse event assessment in TMZ administration according to the CTCAE criteria. [file 41016_2022_271_MOESM4_ESM.docx]

**Table S2** Hematological adverse event assessment in TMZ administration according to the CTCAE criteria

| **Adverse event** | **Reference value** | **Grade 1** | **Grade1/2** | **Grade 2** | **Grade 3** | **Grade 4** | **Grade 5** |
| --- | --- | --- | --- | --- | --- | --- | --- |
| **Leukopenia (10^9^/L)** | 4.00-10.00 | 5.00-5.70 | 3.00-5.00 | 2.00-3.00 | 1.00-2.00 | Aplastic persistent for longer than 2 weeks | Death |
| **Erythropenia (10^12^/L)** | 3.50-5.50 | 2.75-4.13 | 2.63-2.75 | 1.75-2.63 | 0.88-1.75 | Aplastic persistent for longer than 2 weeks | Death |
| **Anemia (g/L)** | 110-160 | 100-110 | - | 80-100 | < 80 | Life-threatening consequences; urgent intervention indicated | Death |
| **Thrombocytopenia (10^9^/L)** | 100-300 | 150-225 | 70-150 | 50-70 | 25-50 | Aplastic persistent for longer than 2 weeks | Death |

* Grade 1 to 5 indicates mild adverse effects, moderate adverse effects, severe or medically significant adverse effects, life-threatening adverse effects and death related to adverse effects, respectively.
