# Supplementary material for: Long-term adjuvant administration of temozolomide impacts serum ions concentration in high-grade glioma
Source: Chin Neurosurg J. 2022 Feb 25;8:6. doi: 10.1186/s41016-022-00271-7 (PMC8876447; doi:10.1186/s41016-022-00271-7)
Supplement: Supplementary file 5 — Additional file 5: Table S3. The grade of adverse events according to CTCAE criteria. [file 41016_2022_271_MOESM5_ESM.docx]

**Table S3** The grade of adverse events according to CTCAE criteria

| **Adverse event** | **Grade 1** | **Grade1/2** | **Grade 2** | **Total (n=73)** |
| --- | --- | --- | --- | --- |
|  | **No.** | **No.** | **No.** | **No. (%)** |
| **1st-3rd Chemotherapy** |  |  |  |  |
| Leukopenia | 34 | 32 | 1 | 67 (91.8) |
| Erythropenia | 14 | 0 | 0 | 14 (19.2) |
| Anemia | 3 | 0 | 1 | 4 (5.5) |
| Thrombocytopenia | 35 | 6 | 0 | 41 (56.2) |
| **4th-6th Chemotherapy** |  |  |  |  |
| Leukopenia | 23 | 39 | 11 | 73 (100) |
| Erythropenia | 13 | 0 | 0 | 13 (17.8) |
| Anemia | 2 | 0 | 0 | 2 (2.7) |
| Thrombocytopenia | 34 | 27 | 0 | 61 (83.6) |
| **7th-9th Chemotherapy** |  |  |  |  |
| Leukopenia | 21 | 38 | 13 | 72 (98.6) |
| Erythropenia | 17 | 0 | 0 | 17 (23.3) |
| Anemia | 1 | 0 | 0 | 1 (1.4) |
| Thrombocytopenia | 34 | 24 | 1 | 59 (80.8) |
| **10th-12th Chemotherapy** |  |  |  |  |
| Leukopenia | 28 | 38 | 4 | 70 (95.9) |
| Erythropenia | 21 | 0 | 0 | 21 (28.8) |
| Anemia | 1 | 0 | 0 | 1 (1.4) |
| Thrombocytopenia | 32 | 21 | 1 | 54 (74) |
